# Why the superb physiological capacity of birds matters

**DOI:** 10.1242/jeb.247986

**Published:** 2025-02-20

**Authors:** Lucy A. Hawkes

**Affiliations:** University of Exeter, Faculty of Health and Life Sciences, Hatherly Laboratories, Prince of Wales Road, Exeter EX4 4PS, UK

**Keywords:** Avian, Migration, Physiology, Physiologging

## Abstract

Among vertebrates, birds undertake the longest, fastest and highest migrations of any taxonomic group, largely due to their unique cardiorespiratory system, which permits for very large rates of gas exchange. Managing resultant elevated production of reactive oxygen species, and thus oxidative stress, has meant that birds can largely avoid pathologies relating to major medical challenges that now probably account for the majority of global healthcare spending. Hypoxia underlies most critical illnesses faced by humans, but the avian cardiorespiratory system can supply far more oxygen per unit of time than any mammal. Birds have high circulating glucose levels, but have adaptations to cope with the elevated production of oxidative stress brought about by hyperglycaemia. Birds also avoid the inflammatory responses brought about by obesity in humans when they seasonally gain huge fat stores. Lastly, birds live four times longer than similarly sized mammals, with seasonal endogenous muscle hypertrophy, and some birds even increase telomere length with age. A new frontier of ‘physiologging’ is emerging, making use of technologies for medical use, but that provide novel parameters for better understanding the biomechanics, energetics and ecology of a range of species. These physiologging tools are likely to provide insight into avian physiology, biomechanics and ecology including their ability to spread disease, as well as each of the medical challenges detailed in this Commentary. By virtue of their physiological capacity, the study of avian physiology is a critical area for future discovery and research using applied and interdisciplinary areas of biomechanics, ecology and physiology.

## Teaser

**Summary:** Birds have highly evolved physiology that may be of interest for future medical innovation, including mechanisms of coping with hypoxia, hyperglycaemia, obesity and age-related sarcopenia, four of the biggest human healthcare challenges.

## Introduction

**Fig. 1.**
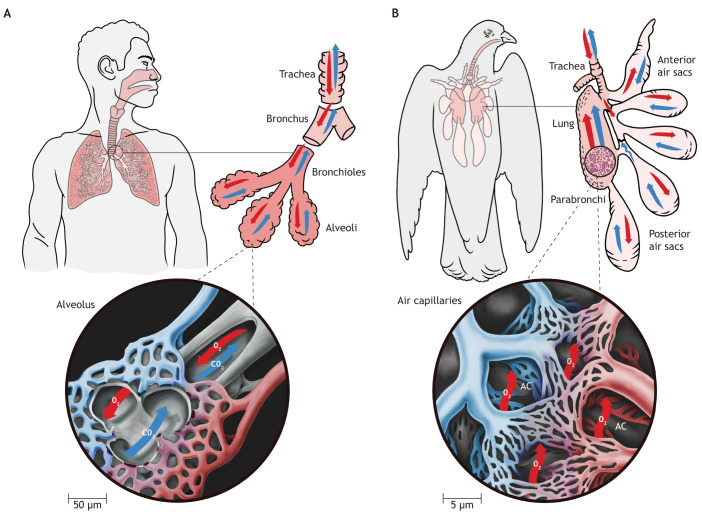
**Drawing showing the comparative anatomy of the human/mammalian and avian respiratory system.** (A) The mammalian lungs are subdivided into bronchioles and terminal alveoli. Magnified inset shows an alveolus surrounded by a capillary network, where red arrows and shading indicate oxygenated air (in the alveolus) and blood (in capillaries) flowing in, and blue arrows indicate deoxygenated air and blood flowing out in the opposite direction. (B) The relatively small avian lungs are supported by a system of air sacs (palest pink) distributed through the birds’ body, which function to generate a unidirectional flow of air through the lung, which is subdivided into parabronchi and air capillaries (right hand side, note red and blue arrows flowing in same direction). Magnified inset shows the rigid avian lung subdivided into air capillaries (AC), which are approximately 10 times smaller than a mammalian alveolus, through which the avian pulmonary capillaries form an enmeshed network with cross-current gas exchange. Scientific illustration by J. Spahr of SciVisual.com.

The most spectacular migrations in the animal kingdom are arguably those carried out by birds; migratory flights take birds over every part of the planet, from the most distant oceans to the highest mountain ranges. This is in large part possible owing to the unusual arrangement of the avian pulmonary system, which facilitates high rates of oxygen uptake. Originally appearing in theropods (from which neoaves are descended), the lung–air sac system likely evolved in response to severe hypoxic conditions in the Mesozoic era (∼250 million years ago; Codd et al., 2008; O'Connor and Claessens, 2005). The Permian–Triassic boundary mass extinction event saw the extinction of 90% of life on Earth, along with a reduction in global atmospheric oxygen to just 11–15% (Berner et al., 2007). During this period, the strong selection pressure for efficient use of oxygen apparently favoured a change in body plan, brought about by a dramatic reduction in genome size, particularly of repetitive and non-coding sequences (modern avian genomes are about a third of the size of mammalian ones, and volant animals – pterosaurs, and later birds and bats – all share small genomes; Nabi et al., 2021; Satoh, 2021). Unlike mammals, the avian respiratory system is composed of relatively small and rigid lungs fixed into the ribs, which are supported by an extensive system of thin-walled and poorly vascularised ‘air sacs’ that are distributed throughout the body from the abdomen to the neck (Fig. 1) (Maina, 2015; O'Connor and Claessens, 2005). During a ventilatory cycle, the air sacs act as bellows to pull and push air unidirectionally through the lung via a series of aerodynamic valves (Butler et al., 1988; Cieri and Farmer, 2016), with air moving from the posterior to the anterior of the bird. The result is that a fresh supply of oxygen moves across the lung surface area during exhalation as well as inhalation, leading to rates of oxygen extraction that are at least twice that of mammals of a similar mass (Cieri and Farmer, 2016; Maina, 2008). The gas exchange anatomy of the avian respiratory system is also more intensely subdivided than that of mammals, and instead of mammalian terminal alveoli, the avian lung divides into air capillaries nearly 10 times smaller than an alveolus, which run cross current to blood capillaries for maximal gas exchange (Fig. 1; Maina, 2008). This has helped birds to become masters of long-distance movement, with flight permitting birds to travel at far greater sustained speeds, and cover far greater distances, than any other means of locomotion. For example, the arctic tern migrates between the Arctic and Antarctica every year (Alerstam et al., 2019), and wandering albatross may travel in excess of 8.5 million kilometres over their >50-year lifetime (Weimerskirch et al., 2014).

Ultimately, this superior locomotory capacity and highly evolved physiology means that birds provide a fascinating lens through which to integrate the study of biomechanics, physiology and ecology. They are a superlative example of the interplay between form, function and performance. The study of avian biomechanics requires an understanding of the physiological limits to which a bird can use theoretical muscular force; the study of avian ecology requires an understanding of the physiological ability of birds to cope with, or move to, favourable conditions to maximise energy intake. Therefore, in this Commentary, I will discuss some areas for future investigations of avian physiology, as well as the potential for inter-disciplinary translation of medical wearable technology into animal science to achieve this. This Commentary is not intended to be a review of avian physiology (instead, see Butler, 2016; Pierce and McWilliams, 2014; Scanes, 2020; Scott, 2011), but I will detail four areas of avian physiology that are of great relevance for future human healthcare challenges, and which have been poorly synthesised to date: hypoxia, hyperglycaemia, obesity, and how birds appear to age in unusually good health, at least compared with what we know of mammals.

## Avian physiology can inform biomedicine

As above, and as I will detail further, avian anatomy and physiology are fundamentally different to that of mammals in many important ways. However, the underlying cellular and molecular processes of disease are conserved across both birds and mammals, and thus the study of bird physiology can generate new hypotheses for human research, inspire innovative approaches to health challenges, and provide a broader context for understanding human physiology and disease. Wild birds are animals that, over the course of a year, and for every year of their athletic lives, experience significant fat gain and loss, live chronically with very high blood glucose, cope with extremes of weather, including environmental hypoxia for some species, and maintain relatively expensive migratory habits.

### Hypoxia

Defined as a deficit of available oxygen, either in the environment or in body tissues, hypoxia is a challenge for aerobic metabolism. The impressive oxygen acquisition ability of the avian lung means that all birds retain a superior capacity for aerobic metabolism in hypoxic conditions compared with mammals (Butler, 2016). In a classic study, Tucker (1968) showed that white mice exposed to a simulated altitude of 6100 m became ‘moribund’, whereas house sparrows could maintain normal rates of activity, and even fly short distances in a hypobaric wind tunnel. In addition to the avian lung–air sac system, all birds (including non-volant birds) have adaptations throughout the oxygen transport cascade that promote oxygen acquisition, including larger hearts and stroke volume, greater capillarity of cardiac and skeletal muscle, and enhanced tolerance of hypercapnia (Butler, 2016). Some bird species have even greater capacity still to tolerate environmental hypoxia. Arguably the best studied is the bar-headed goose (*Anser indicus*), which was reportedly sighted flying over Mount Everest (reviewed in Butler, 2010; Hawkes et al., 2017; Scott et al., 2015), though a range of ducks, geese and swans have also been tracked making passage through the high Himalayas (Damba et al., 2021; Meng et al., 2021; Parr et al., 2019; Xi et al., 2021; Zhang et al., 2020). After thousands of years of exposure, human physiology has been able to adapt to living in chronically hypoxic environments as high as 4500 m (i.e. Tibetan sherpas, Ethiopian highlanders, and populations living on the Andean Altiplano; Beall, 2006; Horscroft et al., 2017), and many of the physiological strategies used by high altitude native people share similarities with high altitude birds (e.g. relative increases in resting total ventilation and altered haemoglobin affinity). Recent work (e.g. Ivy et al., 2019; Laguë, 2017; Laguë et al., 2020; Parr et al., 2017; York et al., 2017; Milsom et al., 2021) has highlighted differing coping strategies by different bird species (Ivy and Guglielmo, 2023). Likewise, strategies used by Tibetan and Andean high altitude native peoples differ (Beall, 2007), showing that although our shared evolutionary routes diverged many millions of years ago, environmental forces can yield similar physiological solutions.

Avian physiology is therefore usually able to maintain tissue oxygenation even in highly hypoxic environments. A special case can be made for penguins, however, and some other diving bird species such as sea ducks, which experience tissue hypoxia via apnea. The emperor penguin dives to 100–500 m (maximum 564 m) and can stay underwater for more than 20 min (Wienecke et al., 2007). In some dives, muscle blood flow may be completely cut off, and ischemic tissues accumulate lactate throughout relatively short dives (∼4 to 5 min; Williams et al., 2011). This strategy may help to maintain oxygen supply to the penguin's brain and heart, and dives are ended when the muscle oxygen stores are depleted. The mechanisms with which penguins (and other diving mammals such as seals) avoid muscle damage during ischemia are not known, though they are relevant to rhabdomyolysis. In humans, rhabdomyolysis is a relatively common pathology during major trauma injuries such as crush syndrome, and can be detected via elevated levels of muscle-type creatine kinase. Future work to measure creatine kinase levels in penguins would therefore be informative. Penguins also face a unique challenge as the relatively rigid avian lung cannot compress or collapse at depth, as deep diving mammalian lungs do to avoid barotrauma, and penguins maintain gas exchange in the air capillaries during diving (Ponganis, 2025). Interestingly, continued movement of air around the lung, possibly funded by underwater wing beats, is analogous to ‘apneic oxygenation’ used in some hypoxaemic human patients during surgery (Ricard, 2016; Ponganis, 2025). Finally, because they do not have the need for large rates of oxygen consumption for flight, the avian lung may thus actually acquire excess oxygen for penguin life, such that counter-adaptations such as reduced lung size and thicker blood–gas barriers may have evolved in penguins to reduce gas diffusing capacity to just 15% of flighted birds (Ponganis, 2025). Oxygen deprivation fundamentally underlies almost every critical illness faced by humans, including cardiac disorders, strokes and other vascular anomalies, respiratory ailments, sepsis and trauma. These vascular, metabolic and cellular mechanisms that birds use to cope with hypoxia have not been considered within the realm of medical innovation. Here, I suggest there is a rich potential for translational medicine to take inspiration from avian physiology.

### Hyperglycaemia

A perhaps surprising adaptation that evolved at the same time as the development of the lung–air sac system in theropods (Codd et al., 2008; O'Connor and Claessens, 2005) was insulin resistance (Satoh, 2021). Insulin resistance may be helpful in hypoxic conditions by maintaining a net mobilisation of glucose for metabolism, by promoting use of alternate fuels such as fats, and by reducing the cost of conversion of glucose into stored energy (e.g. glycogen and triglycerides). In comparison with mammals of the same size, birds have approximately two to four times higher circulating levels of blood glucose, but live long, healthy lives apparently free from pathologies that are typically associated with hyperglycaemia in humans (the ‘bird paradox’; reviewed in Braun and Sweazea, 2008; Sweazea, 2022). In mammals, high levels of glucose in the blood stimulate the release of insulin, promoting the absorption of glucose from the blood into the liver (i.e. insulin promotes glucose homeostasis). Diabetes results from insufficient insulin production by beta cells in the pancreas (Type I diabetes), or resistance/insensitivity to insulin (Type II diabetes), resulting in hyperglycaemia (and/or uncontrolled hypoglycaemia). In humans, diabetes is associated with lethargy, weight gain, neuropathy (nerve damage), ketonuria (increased production of ketone bodies), excess production of cytokines, and oxidative stress (Antar et al., 2023; Brownlee, 2001; Yagihashi et al., 2011). Diabetes is one of the leading causes of death and disability worldwide, affecting 6% of the global population in 2021, and predicted to rise to over 10% by 2050 (Ong et al., 2023).

Although the avian pancreas does produce insulin, and the avian liver is sensitive to it, it has been proposed that birds are largely insulin-resistant (akin to Type II diabetes), with very limited insulin-mediated glucose uptake in fat and skeletal muscle tissue, and a paucity of insulin receptors (Dupont et al., 2012; Sweazea, 2022). Pancreatectomy does not generally result in dysregulation of glucose in birds (Sitbon et al., 1980). Birds also rarely express the functional insulin-responsive glucose transport protein GLUT-4, which is fundamental in mammalian glucose uptake into skeletal muscle (Sweazea, 2022). Two major questions then arise from this peculiarity: (i) why are birds chronically hyperglycaemic; and perhaps more importantly, (ii) how do they tolerate hyperglycaemia?

First, it is tempting to suggest that because the energetic costs of flight are high compared with those of swimming and running (Butler, 2016), the surplus glucose is used for ATP production. This appears largely not to be the case, with the majority of energy production (∼90%) for flight likely funded by fatty acid metabolism, which yields a greater return of ATP per mole of fuel (Guglielmo, 2018; Jenni-Eiermann, 2017). Birds store almost all their circulating glucose in plasma (mammals store 50% in plasma and 50% in erythrocytes) (Jenni-Eiermann, 2017), but owing to their lower energy density, even high quantities of circulating glucose and glycogen are insufficient to fuel flights of the lengths and distances observed in wild birds (Guglielmo, 2018). More likely hyperglycaemia in birds results from a lack of need to regulate glucose at lower levels.

Second, therefore, birds can tolerate hyperglycaemia owing to adaptations to manage reactive oxygen species (ROS) and subsequent oxidative stress, which are responsible for the majority of diabetes pathology in humans, i.e. hyperglycaemia does not incur a physiological cost in birds as it does in mammals (Vágási et al., 2024). Indeed, ROS plays a central role in the pathology of some of the most significant diseases in humans, including cardiovascular disease, neurodegenerative diseases such as Alzheimer's and Parkinson's, cancer, autoimmune diseases and metabolic disorders (Liu et al., 2018). In birds, adaptations to manage ROS include first decreasing ROS production, which they achieve via increasing uncoupling of mitochondria (the ‘uncouple to survive’ hypothesis; Brand, 2000), which also increases thermogenesis. Humans express uncoupling proteins (UCP1, 2 and 3) in various tissues, and the relative expression of these proteins increases with exercise, but decreases with age and body fat index (Hirschenson et al., 2022). UCP1, for example, is expressed in brown fat to promote non-shivering thermogenesis of humans in cold environments. However, in modern society, characterized by an aging population and increasingly poor diet and sedentary lifestyles, factors that instead suppress UCP expression have become more prevalent. Nevertheless, therapeutic strategies promoting UCP expression may be worth future investigation. In response to reducing ROS production, birds can also increase expression of antioxidants including uric acid, glutathione, vitamin C (which birds can endogenously synthesise) and superoxide dismutase (Cohen et al., 2007), as well as other antioxidant enzymes (Kasai et al., 2020). In contrast, humans get the majority of antioxidants via a diverse and healthy diet (Liu et al., 2018). Birds can also increase mitochondrial oxidative capacity, without also increasing ROS production, via even greater proton leakage at the mitochondria (Coulson et al., 2024). The mechanisms by which hyperglycaemia thus causes, and can be ameliorated by, ROS production and scavenging, are common to both birds and humans. Some novel potential treatment pathways for humans seek to understand the molecular pathways leading to inflammation and oxidative stress caused by diabetes (Antar et al., 2023). I suggest that the ways in which birds cope with hyperglycaemia could inspire this, provide new targets for drug development, and/or provide insights for preventing diabetes complications. Finally, how glucose is modulated, transported and stored, as well as how ROS are produced and diminished during extremes in birds, i.e. during migration, has yet to be studied and is a rich area for future work using medical wearable technologies such as continuous glucose monitors and other enzyme-based sensors.

### Obesity

Of the ∼41 million human deaths from non-communicable diseases each year, approximately 12% are driven by obesity (defined by the World Health Organisation as an excess accumulation of body fat, and a weight to height ratio over 25), costing ∼120 million years of life lost and predicted to rise from 42% (in 2020) to 54% of adults by 2035 (World Obesity Federation, 2024). This is a major global challenge as modern food production is both environmentally damaging (Hall et al., 2009) and costly to global healthcare systems (costing up to 3% of global GDP by 2035; World Obesity Federation, 2024). At the same time, fat is an important tissue, serving a variety of essential functions across taxa (Knight, 2018). Thus, obesity is not particularly rare in wild mammals, with hibernating animals having 30–40% of their body mass as fat prior to torpor (Giroud et al., 2021). In addition, marine mammals, which need large amounts of body fat for buoyancy and insulation from cold waters, sometimes have more than 50% body fat stores (Oller et al., 2021; Slip et al., 1992; Strandberg et al., 2008). Prior to migration, wild birds can gain astonishing amounts of fat, in some cases foraging around the clock to more than double their mass (Guglielmo, 2018; Piersma and Gill, 1998), largely via fat depots (e.g. Vézina et al., 2021). Fat is the consummate fuel for migrating birds because it can hold more than seven times the energy density of glycogen, and eight to 10 times more chemical energy than wet protein or carbohydrates (Guglielmo, 2018; Jenni-Eiermann, 2017). In humans, adipose tissue secretes pro-inflammatory cytokines (adipokines), which lead to oxidative stress and can alter insulin signalling and sensitivity (Wu and Ballantyne, 2020). It is currently unclear whether hypertrophy in avian adipose tissues brings about similar inflammatory responses (Bernardi et al., 2021). One might hypothesise it does not (and may also not in wild mammals such as seals with a higher obesity ‘set point’), and the mechanisms allowing this seem well worth investigation for novel strategies for treating obesity-related pathologies (e.g. Angelidi et al., 2022; Khudyakov et al., 2019).

Birds achieve their extraordinary rate of fat gain (up to 15% of lean body mass per day in the most extreme cases) by increasing the rate at which they feed (typically by 40% more, but up to 500% more than maintenance levels; Guglielmo, 2018). Like humans, they also show a preference for more carbohydrate- and fat-rich food (e.g. fruits and seeds), which can be readily converted to stored lipids (Bairlein, 2002). As in mammals, feeding may be stimulated by increased expression of (and response to) ghrelin (the ‘hunger hormone’) and corticosterone, but whereas mammalian adipocytes produce leptin to regulate (inhibit) feeding, it appears that avian adipocytes do not, and even when experimentally administered, leptin does not always inhibit feeding during the migratory period, and thus may aid excessive fattening (Cerasale et al., 2011; Rossi and Welch, 2023). Obesity in humans is also associated with leptin resistance (Mendoza-Herrera et al., 2021), but it is unclear how birds regulate feeding and consequent fattening outside of the migratory period, and it is possible that other regulatory mechanisms are involved. The latest anti-obesity treatments include drugs simulating the effect of glucagon-like peptides (e.g. GLP-1), which act in the shorter term to suppress appetite, and appear to function similarly in both mammals and birds (Failor et al., 2023).

Finally, the avian digestive system exhibits considerable plasticity – retaining a far superior capacity for passive nutrient absorption than non-volant mammals (Guglielmo, 2018). It is also able to dramatically change in size (along with the liver) during hyperphagia (Guglielmo and Williams, 2003), along with digestive enzymes and intestinal transporters, such as fatty acid synthase, 9-desaturase, glucose-6-phosphate dehydrogenase and malic acid (Guglielmo, 2018). The gut–brain axis (and microbiome) is of considerable human health interest, as digestive disease may underlie a range of health conditions, including autoimmune disorders, metabolic syndrome, nutrient deficiencies and even mental health disorders (Gomaa, 2020).

### Sarcopenia

Sarcopenia is the loss of lean muscle mass and power, and is recognised as an age-related disease that leads to functional decline, frailty (particularly in geriatric people), increased risk of falling and thus increased demand for healthcare (Cruz-Jentoft et al., 2019; Wiedmer et al., 2021). Severe sarcopenia may be present in as much as 10% of older adults (Wiedmer et al., 2021; Yuan and Larsson, 2023). Ageing can be viewed as the result of two components: primary ageing refers to intrinsic physiological degradation over time not related to stress, disease or trauma, whereas secondary ageing occurs as a consequence of extrinsic environment and lifestyle factors such as diet and exercise (Shur et al., 2021). In sarcopenia relating to primary ageing, mechanisms of muscle degradation include decreases in the levels of anabolic hormones (e.g. testosterone, IGF-1 and insulin), a decrease in neuromuscular drive and muscle fibre composition, reduced myo-satellite cell recruitment, impaired mitochondrial function, and increased susceptibility to inflammation and oxidative stress (reviewed in Wiedmer et al., 2021). There is currently no evidence that birds suffer age-related sarcopenia – instead, there are records of very old animals migrating with the rest of their populations (e.g. Laysan albatross 73 years old, Manx shearwater 51 years old, oystercatcher 47 years old, fulmar 45 years old, puffin 45 years old, arctic skua 33 years old; https://euring.org/data-and-codes/longevity-list). By virtue of maintaining migratory habits (i.e. exercise), birds may thus also moderate secondary ageing, along with preferentially ingesting a diet rich in anti-oxidants prior to migration (Alan and McWilliams, 2013; McWilliams et al., 2021). This strongly suggests that age-related sarcopenia may either not occur in birds, or at least may be delayed or occur to a far lesser degree. The relationship between telomere (the protective end cap protecting DNA strands from damage during replication) degradation and cellular senescence suggest an important role of telomere shortening in age-related declines of physiological function. By contrast to mammals, telomere length appears to increase in length with age in a few (but not all) bird species (e.g. oystercatchers and Leach's storm petrels; Tricola et al., 2018). Birds also live approximately four times longer than similarly sized mammals, likely because of their ability to cope with oxidative damage (McWilliams et al., 2021).

In birds, flight muscles increase in anticipation of migration, even in captive birds and in the absence of exercise, in only a few days (Battley and Piersma, 1997; Price et al., 2011; Vézina et al., 2021). For example, the red knot (*Calidris canutus*) may gain as much as 19% lean dry pectoralis muscle mass without exercise (Piersma et al., 1999). The mechanisms underpinning avian seasonal flight muscle hypertrophy vary between species (reviewed in Elowe et al., 2023; Price et al., 2011). Myostatin is a growth inhibitor that is highly conserved across mammals and birds, and promotes quiescence of satellite cells so that they do not proliferate into new muscle fibres (McFarland et al., 2007). Birds likely suppress myostatin, and increase expression of insulin-like growth factor-1 mRNA (Piersma and van Gils, 2011), which is a potent stimulant for skeletal muscle growth (Hennebry et al., 2017; Snijders et al., 2015). IGF-1 expression has been less well studied in wild birds than myostatin, but several studies on captive birds have suggested it is upregulated prior to migration (reviewed in Price et al., 2011). Furthermore, avian muscular hypertrophy may also involve a range of adaptations to improve response to muscle damage, such as inflammatory signalling pathways (Elowe et al., 2023).

Overall, though flight muscle hypertrophy in wild birds has been insufficiently studied, the common mechanistic pathways with humans provide a basis for further study of wild birds toward an applied outcome for healthy ageing across the lifespan. Of considerable interest would be studies of gene expression of markers of muscle atrophy, apoptosis, growth and maintenance, as well as oxidative stress in populations of birds with extended life spans (e.g. nesting fulmars in the Orkney Islands have been studied since 1950). How birds appear to age in good health has important implications for novel ways to manage sarcopenia in humans, including bed rest, frailty and even long-duration spaceflight.

## Implications of avian physiology for disease transmission

The constrained model of total energy expenditure posits that at higher levels of physical activity, compensatory (energetic) savings in reproduction, digestion, growth, somatic maintenance and immune function can be made to minimise total energy expenditure (Pontzer et al., 2016). This has been demonstrated in a range of animals including birds (reviewed in Pontzer, 2015); for example, zebra finches had lower average daily energy expenditure when subjected to higher workloads in captivity (Deerenberg et al., 1998). This is important because it suggests that during migration, despite (or perhaps even because of) their enhanced capacity for high metabolic rates compared with mammals, birds may reduce investment in immune defence, and this has been demonstrated to some extent in passerines and quail (Eikenaar and Hegemann, 2016; Eikenaar et al., 2020; Tobolka et al., 2024). This, coupled with their mastery of long-distance flight, means that the range over which birds can spread disease, compared with other taxa, is exceptional (Klaassen and Wille, 2023). For example, an avian panzootic of unprecedented magnitude emerged in late 2021, and devastated bird populations across Asia (Cui et al., 2022) and North America (Caliendo et al., 2022; Ramey et al., 2022), and seabird colonies in Europe (Lane et al., 2024), and most recently in South America and Antarctica (Gamarra-Toledo et al., 2023; Leguia et al., 2023). In addition, the majority of global avian biomass is made up of poultry (Bar-On et al., 2018), and cycles of disease between wild birds and poultry are likely responsible for the emergence and perpetuation of viral strains (Gilbert et al., 2017). That poultry are also birds, and therefore share the highly evolved physiological features detailed in this Commentary, means that a deep consideration of bird physiology will be fundamental to future management of global zoonoses.

## Tools for the job

**Fig. 2.**
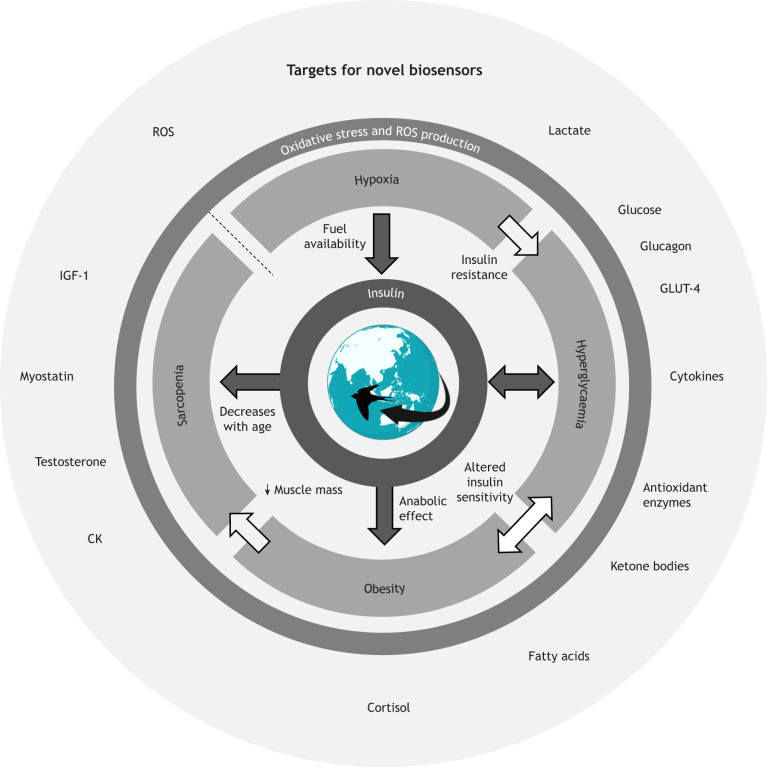
**Schematic diagram showing four major healthcare areas for human medicine (hypoxia, hyperglycaemia, obesity and sarcopenia) and how avian physiology has evolved solutions to all of them that interact.** At the core of these adaptations is a modulation of insulin responsiveness compared with mammals, and effective management of reactive oxygen species (ROS). Potential targets for novel biosensor applications are shown in the outermost ring, following models of wearable continuous glucose monitors (CGMs). CK, creatine kinase. Globe and passerine icons created in BioRender by Hawkes, L., 2024. https://BioRender.com/o32w896. This figure was sublicensed under CC-BY 4.0 terms.

A new frontier of ‘physiologging’ has been emerging over the last decade (Macdonald et al., 2021; Williams et al., 2021), making use of biologging technologies that have been developed for medical use, but that provide novel parameters for better understanding the physiology of a range of species. These seem ideally suited to adaptation for use in wild animals, and the market opportunity is ample for blue skies translational development into wildlife research. For example, the market for continuous glucose monitoring (CGM) devices is predicted to be worth $36.7 billion by 2026 (https://www.marketsandmarkets.com/Market-Reports/biosensors-market-798.html), and the state of the art (for commercially available devices) includes CGMs that weigh just 4 g (i.e. small enough to be deployed on thousands of bird species). These devices include a waterproof housing, and can collect data on glucose levels for up to 14 days at 15-min resolution. The largest market share of CGMs are enzyme-based biosensors, which directly measure electrochemical oxidation of a target molecule by an enzyme, which is embedded in a needle electrode. Such sensors for glucose have existed since the 1960s, but novel biosensors are entering the market all the time (e.g. sensors for lactate, alcohol, uric acid, glutathione and vitamin C; Bucur et al., 2021; Cheraghi et al., 2022; Sempionatto et al., 2020). Developing biosensors to detect target molecules such as creatine kinase, UCP, ROS and different cytokines could provide valuable insights into avian physiological mechanisms across the four major human health areas covered in this Commentary (Fig. 2). This would enhance our understanding of how external factors, such as disease, impact avian biology in the wild.

## Conclusions

The unique respiratory system and evolved mechanisms to manage oxidative stress of birds not only enable their impressive long-distance migrations but also offer valuable insights for medical research. The suggestion of extreme animals as models for human therapeutic interventions is not new (Bertile et al., 2021; Fröbert et al., 2020; Oller et al., 2021; Sweazea, 2022), but it is noteworthy that birds have seemingly found solutions to many of society's most pressing human healthcare issues – perhaps more so than any other taxonomic group. The superb physiological capacity of birds underscores their critical importance in biomedicine, disease spread and more. As we look to the future, key next steps will be to integrate this understanding into broader biomechanical and ecological models. By continuing to learn from birds, we may not only increase our appreciation for them, but also pave the way for advances in human health and wellbeing. Future important scientific advances may come from most unexpected places – in this case the skies above us.
